# Proposed guidelines on the evaluation of non-antibiotic versus antibiotic agents indicated for treatment of uncomplicated acute cystitis in adult female patients

**DOI:** 10.3205/id000104

**Published:** 2026-01-28

**Authors:** Kurt G. Naber, Jakhongir F. Alidjanov, Adrian Pilatz, Florian M. Wagenlehner

**Affiliations:** 1 Department of Urology, Technical University of Munich, Germany; 2 Biostatistics & Data Science, Nuremberg, Germany; 3 Clinic for Urology, Pediatric Urology and Andrology, Justus-Liebig University of Giessen, Germany

## Abstract

Since several current clinical guidelines also recommend non-antibiotic therapy of uncomplicated acute cystitis (uAC) in women, research guidelines are needed on conducting clinical trials to demonstrate their efficacy compared to e.g. standard antibiotic therapy. As the mechanism of action of antibiotic and non-antibiotic therapy is different, clinical outcome in such comparative trials must be the main criteria, although the effect of microbiological outcomes on clinical outcome may also be considered. The research guidelines proposed here are adjusted to the current guidelines recommended by the European Medicines Agency (EMA, 2022) and the U.S. Food and Drug Administration (FDA, 2019), using in addition a patient self-reporting questionnaire already clinically validated in many languages for diagnostics of uAC and as a patient-reported outcome measure (PROM) with well-defined thresholds for successful and non-successful clinical outcome. These adapted guidelines could be used in prospective clinical studies comparing e.g. antibacterial and non-antibacterial products for the treatment of women suffering from uAC.

## 1 Introduction (Background)

In the face of increasing problems caused by emerging bacterial resistance, there is an urgent need to develop non-antibacterial agents suitable for treating women with uncomplicated acute cystitis (uAC) to minimise the risk of selecting for bacterial resistance. Since in the past treatment of uAC with antibiotics was considered the standard, the efficacy of non-antibacterial agents should be evaluated in comparison with antibiotic treatment as recommended by international guidelines.

Although current guidelines recommend antibiotics as the first choice of treatment for the acute phase of cystitis [1], [2], [3], several prospective randomized, placebo-controlled studies comparing antibiotic and non-antimicrobial symptomatic therapeutic modalities have been performed [4], [5], [6], [7].

To facilitate clinical development programmes also for non-antibacterial agents and to support modifications to the uses and/or regimens for licensed agents, there is a need for accepted guidelines on how to perform clinical studies to demonstrate their efficacy as compared to e.g. standard antibiotic therapy and to ensure that each clinical trial conducted also meets the requirements of multiple regulatory agencies.

## 2 Scope

Since the therapeutic effect with antibacterial agents and non-antibacterial agents is different in principle, well-defined inclusion and outcome criteria need to be agreed on to compare the therapeutic results of both treatment modalities. Whereas antibacterial agents are administered because of their bactericidal or bacteriostatic effect on the causative uropathogens, non-antibacterial agents may only reduce some virulence properties of the uropathogens, e.g. their adhesion on epithelial cells, or act only on the host response, e.g. anti-inflammatory effect. For scientific reasons it would be desirable to investigate the effect of all such therapeutic principles in both treatment arms: 1) elimination of bacteriuria and 2) host inflammatory response, e.g. leukocyturia, cytokines, etc.

Whenever patients were included with the appropriate diagnosis of uAC, the early and late clinical outcome, measured as the patient-reported outcome (PROM), determined by appropriate tools is finally crucial.

The proposed guidelines on how to perform clinical studies comparing antibiotic with non-antibiotic agents in the treatment of uAC were developed also considering the general principles as published in the updated guidelines according to the European Medicines Agency (EMA) [8] and the U.S. Food and Drug Administration (FDA) [9].

## 3 Patient selection

According to EMA guidelines 2022 [8], female patients with uAC should have a minimum number of symptoms such as frequency, urgency and dysuria. Patients may be enrolled before microbiological culture results are available based on documented pyuria (≥10 WBCs/mm^3^) in a mid-stream specimen.

According to the FDA guidelines [9], the inclusion criteria for patient selection are fairly similar: patients should be adult females and, if appropriate, adolescent females with evidence of pyuria and at least two of the following signs or symptoms of uncomplicated urinary tract infection (uUTI): dysuria, urinary frequency, urinary urgency, suprapubic pain.

Since patient selection criteria should maximize the likelihood that patients under study have indeed uAC before microbiological culture results are also available, validated questionnaires could be used, such as the Acute Cystitis Symptom Score (ACSS) [10].

The ACSS is a patient self-reporting questionnaire consisting of two parts: diagnostic Part A and follow-up Part B. Each part contains 18 items, allocated to four domains: six items on typical symptoms of uAC (“Typical” domain), four items for differential diagnosis (“Differential” domain), three items on quality of life (“QoL” domain), and five items on additional conditions that may affect therapy (“Additional” domain”). Each item of the first three domains (“Typical”, “Differential” and “QoL”) is fitted with a 4-point Likerttype scale for assessing the severity of each symptom ranging from 0 (no symptom or discomfort) to 3 (severe symptom or discomfort). The “Additional” domain contains dichotomous “yes/no” questions. Furthermore, Part B includes an additional “Dynamics” domain formed by a question about the general evolution and changes in symptoms (see Attachment 1; the questionnaire, including versions in other languages, is also available at https://www.acss.world).

To develop suitable inclusion criteria we used the data of a clinical study in which 517 female respondents (285 patients with uAC and 232 controls without uAC) derived from the e-USQOLAT database were included [11]. Only cases with sufficient information concerning ACSS data and urinalysis were selected for further statistical analysis. The diagnosis concerning the presence or absence of uAC made by the treating physician based on the history and the results of the laboratory findings in accordance with national and/or international standards and guidelines [1], [2], [3] was taken as reference.

In this study [11] it could be shown that not only the presence but also the severity of the symptoms is important because all of the so-called “typical symptoms”, such as urinary frequency, urinary urgency, dysuria, suprapubic pain, and sense of incomplete bladder emptying, do not differentiate significantly between patients and controls if these symptoms are only present in a mild form.

Only the presence of mild visible blood in urine, which corresponds to the 6^th^ symptom of the ACSS, could differentiate significantly between patients and controls also if present in a mild form only.

In Table 1 (Tab. 1) we demonstrated several diagnostic parameters, such as sensitivity, specificity, positive and negative predictive values (PPV, NPV), positive and negative likelihood ratio (+LR, –LR), diagnostic odds ratio (DOR), Youden’s index, area under the receiver operating characteristic (ROC) curve (AUC), correlation with positive outcome (PO) for diagnosis of uAC according to the number and severity of symptoms with and without presence of pyuria. The first three symptoms are mentioned in the EMA guidelines, the first four symptoms in the FDA guidelines and the five symptoms and in addition “visible blood in urine” in the ACSS. If the summary score of the symptoms is taken so that the severity of at least one symptom must be more than mild, it can be shown that the average sensitivity ranged between 0.84 and 0.87 and the specificity between 0.88 and 0.89 for correct diagnosis of uAC. The highest value of Youden’s index and AUC represent the best balance between sensitivity and specificity found for the results of the ACSS.

Positive pyuria by itself possesses a sensitivity of 0.85, but the specificity is much lower: at 0.72. If, however, a positive pyuria test is combined with the presence of symptoms, the average sensitivity ranges between 0.70 and 0.73 and the specificity between 0.96 and 0.97. The highest Youden’s index accompanied by the highest AUC was again found for the ACSS’s pre-defined cut-off value. Although it has been demonstrated that the scoring of the five first typical symptoms in the ACSS is not much inferior to the six symptoms, including visible blood, we recommend further to include all six items in the typical domain because visible hematuria in connection with typical urinary symptoms may be pathognomonic for acute hemorrhagic cystitis. It can also be an important differential sign. If visible hematuria persists after treatment, it needs a further careful investigation of the patient to exclude any other urological disease, such as bladder cancer.

Therefore, for patient selection for a comparative study with antibacterial and non-antibacterial agents in the treatment of uAC in women, we recommend to include only those patients with documented pyuria (≥10 WBCs/mm^3^) in a mid-stream urine specimen as also recommended by the EMA and FDA guidelines and a minimum summary severity score related to the considered number of typical symptoms. The best balance between sensitivity and specificity with the highest Youden’s index and AUC was found when using a minimum summary score of 6 for the “Typical domain” using the ACSS.

## 4 Microbiological investigations

Since antibacterial agents recommended by updated international guidelines, e.g. European Association of Urology (EAU) guidelines on urological infections, should be used as comparative agents to investigate the efficacy of the non-antibacterial agents to be tested, all recommended microbiological investigations should be performed in urine cultures for diagnostics and patient-reported outcome in both groups as well. However, the use of a general definition for significant bacteriuria of ≥10^5^ CFU/mL as an inclusion criterion for the microbiological intention to treat (ITT)-population (EMA guidelines 2022) may falsely exclude about half of the patients with uAC presented with about the same severity of symptoms. Bacteriuria of ≥10^5^ CFU/mL in adults was originally defined as significant only for the diagnosis of pyelonephritis [12].

In 1982, Stamm et al. [13] documented that the levels of ≥10^5^ CFU/mL of a pathogen in urine have a very high specificity (99%) but a very low sensitivity (51%) for the diagnosis of uAC. Bacteriuria of ≥10^2^ CFU/mL was suggested by the authors as the best diagnostic criterion (sensitivity, 95%; specificity, 85%). In 2013, Hooton et al. [14] confirmed that *Escherichia coli* identified as low as 10^1^–10^2^ CFU/mL was sensitive and specific for the diagnosis of uAC in symptomatic women, but still about 20% of these symptomatic female patients were culture “negative” even when being tested for such low counts. Quantitative polymerase chain reaction (qPCR) for *E. coli* and *S. saprophyticus* finally demonstrated that almost all women with symptoms suggestive of UTIs and a “negative” culture still had an infection with *E. coli* [15].

Therefore, according to the German National S3 Guideline, the detection of *E. coli* in symptomatic women is predictive for a bacterial UTI, irrespective of the number of pathogens. In contrast, the presence of *Enterococci* and group B *Streptococci* in urine is not predictive for UTIs [16].

## 5 Outcome

At best all patients included into the study should be evaluated concerning outcome at several times, e.g. for early effect at about 2–4 days after start of therapy, at end of therapy, at test of cure (TOC), about 5–7 days after end of treatment, and follow-up, about 3–4 weeks after end of treatment. At each visit careful evaluation of still present symptoms including their severity, change in QoL and possible side effects needs to be performed. Always using the same questionnaire has the advantage that the patient may have developed an internal standard about the appearance and severity of the different symptoms asked for at each visit in the same format.

Although microbiological investigations in urine should be performed not only at the first visit before therapy, but at least in case of clinical failure at any time and routinely at TOC and at follow-up about 3–4 weeks after TOC in the microbiological ITT-population, the primary analysis should be the clinical outcome at all follow-up visits. If antibacterial and non-antibacterial agents are compared in their efficacy, elimination of bacteriuria as the main study aim is scientifically questionable due to the findings that asymptomatic bacteriuria may probably even be protective against recurrent UTI [17].

How to define best thresholds for clinically successful (cure) and non-successful outcome (failure) could be developed from a clinical study [18]. Data from 134 female patients with diagnosed uAC were included in the current analysis with (1) a summary score of “Typical” domain of 6 and more; (2) at least one follow-up evaluation after the baseline visit; (3) no missing values in the ACSS data.

Six different predefined thresholds based on the scoring of the ACSS items were evaluated to define best “clinical cure”, also considering the FDA and EMA guidelines.

Table 2 (Tab. 2) shows the patient-reported-outcome (PRO) during an early follow-up visit (2–4 days after start of treatment), at end of treatment (5–9 days after start of treatment), and at a late follow-up visit (10–30 days after end of treatment) with clinical successful outcome (n, %) according to five different thresholds [18]. A summary score of the five typical symptoms of ACSS of ≤5 with no symptom more than 1 (mild) and without visible blood in urine was favoured, although the results obtained with the three or four symptoms mentioned by EMA [8] and FDA [9] guidelines, respectively, used in a similar way also show very similar results at end of treatment and late follow-up. Therefore, it would be recommended to use one of these threshold criteria for the study protocol. If QoL criteria are added to the evaluation of the ACSS typical symptoms, the results of successful clinical outcome are somewhat reduced. The overall patient’s clinical assessment (“Dynamic” domain) alone was not sensitive enough in our opinion for a suitable PRO measure [18].

In clinical studies also using non-antibacterial agents as comparator the clinical outcome needs to be considered as primary outcome in the clinical ITT-population at TOC using a non-inferiority margin of –10%, whereas the clinical outcome at late follow-up, usually 3–4 weeks after TOC, could add good information on the relapse or reinfection rate. Therefore, it is important that clinical success and failure are clearly defined in the study protocol. All patients judged by the treating physician or by the patient herself to consider the present therapy as insufficient and switching to (another) antibiotic therapy need to be classified as clinical failure as well. In addition, using a validated questionnaire asking for typical symptoms of uAC and their severity as performed in the ACSS [10], a summary score of the 5 ACSS typical symptoms ≥6 (or the 4 FDA typical symptoms ≥5 or the 3 EMA typical symptoms ≥4) or any of the typical symptoms with a severity of at least 2 need to be considered as clinical failure as well. Persistent visible blood in urine may be also a clinical failure, but in case of persistent visible blood in urine (of any severity) there is also a great suspicion that the source of the bleeding may not be due to the present infection and therefore differential diagnostic clarification may be required.

Although microbiological outcome may not be included in the primary analysis, it would be of great interest to evaluate microbiological outcome in the ITT-population as well, e.g. to investigate in which therapeutic format the eradication or persistence of uropathogens is correlated with clinical outcome at TOC and at late follow-up. In patients treated with non-antibacterial agents their efficacy may not always be correlated with eradication of bacteriuria. As mentioned earlier, asymptomatic bacteriuria may even be protective against recurrent UTI [17]. Therefore, the late follow-up visit after about 4 weeks (24–33 days) after start of treatment should be performed on all patients with clinical success at test of cure visit without but also with bacteriuria (asymptomatic bacteriuria). In this case the role of asymptomatic bacteriuria could be tested concerning relapse and recurrence until late follow-up visit in those patients treated with antimicrobials and non-antimicrobials.

The assessment and presentation of the safety data should follow the recommendations proposed by EMA in their guideline on the evaluation of medicinal products indicated for treatment of bacterial infections [8]. In Table 3 (Tab. 3) the proposed guidelines are summarized as a decision tree showing which actions are needed at which stage of such a prospective, randomised, double-blind, placebo controlled study on the evaluation of non-antibiotic versus antibiotic agents indicated for treatment of uAC in adult female patients.

## 6 Conclusions

In clinical studies comparing antibacterial and non-antibacterial agents to treat uAC the clinical outcome needs to serve as primary analysis, although microbiological outcome should also be evaluated as well. To improve clinical outcome, criteria for scoring the severity of so-called typical symptoms of uAC are needed not only for diagnosis, but also for patient-reported-outcome (PRO) to define a threshold for successful clinical outcome (clinical cure) of any intervention, which could be combined with QoL issues and overall outcome assessed by the patients. In clinical studies it could be demonstrated that the ACSS has the potential to be used as a suitable diagnostic and PRO measure and should therefore be recommended for standard guidelines in prospective clinical comparative studies using antibacterial and non-antibacterial products for treatment of women suffering from uAC.

## Note

This article is also to be published as a chapter of the Living Handbook “Urogenital Infections and Inflammations” [19].

## Copyright of the ACSS

The ACSS is copyrighted by the Certificate of Deposit of Intellectual Property in Fundamental Library of Academy of Sciences of the Republic of Uzbekistan, Tashkent (Registration number 2463; 26 August 2015) and the Certificate of the International Online Copyright Office, European Depository, Berlin, Germany (Nr. EU-01-000764; 21 October 2015). The rightsholders are Jakhongir Fatikhovich Alidjanov (Uzbekistan), Ozoda Takhirovna Alidjanova (Uzbekistan), Adrian Martin Erich Pilatz (Germany), Kurt Guenther Naber (Germany), and Florian Martin Erich Wagenlehner (Germany). The e-USQOLAT is copyrighted by the Authorship Certificate of the International Online Copyright Office, European Depository, Berlin, Germany (Nr. EC-01-001179; 18 May 2017). Translations of the ACSS in other languages are available on the website https://www.acss.world/downloads.html.

## Authors’ ORCIDs

- Kurt G. Naber: 0000-0003-1304-5403
- Jakhongir F. Alidjanov: 0000-0003-2531-4877
- Adrian Pilatz: 0000-0001-8072-1841
- Florian M. Wagenlehner: 0000-0002-2909-0797

## Competing interests

KGN, JA, AP, and FMW are authors and copyright holders of the ACSS. KGN is a consultant of Adamed Pharma, Bionorica, BioMerieux, GlaxoSmithKline, Immunotek, Ingenion Medical, Johnson & Johnson, OM Pharma, and MIP Pharma. JA is an employee of Bionorica SE. FMW is a consultant of Achaogen, Astellas, AstraZeneca, Bionorica, MSD, Eumedica, GSK, Janssen, Klosterfrau, MIP Pharma, Pfizer, OM Pharma, Qiagen, VenatoRx. 

## Supplementary Material

American English Acute Cystitis Symptom Score (ACSS) – Questionnaire

## Figures and Tables

**Table 1:**
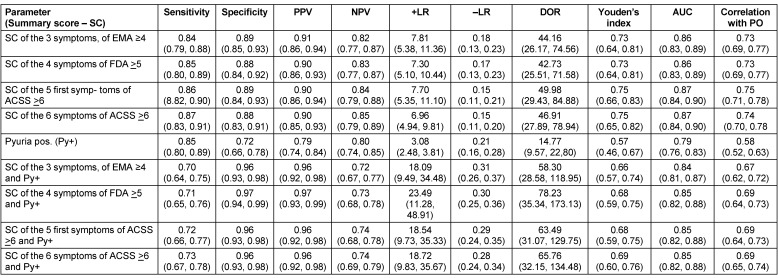
Sensitivity, specificity, positive and negative predictive values (PPV, NPV), positive and negative likelihood ratio (+LR, –LR), diagnostic odds ratios (DOR), Youden’s index, area under the curve (AUC), correlation with positive outcome (PO) for diagnosis of uAC according to the number of symptoms. Average value [95% confidence interval] [11]

**Table 2:**
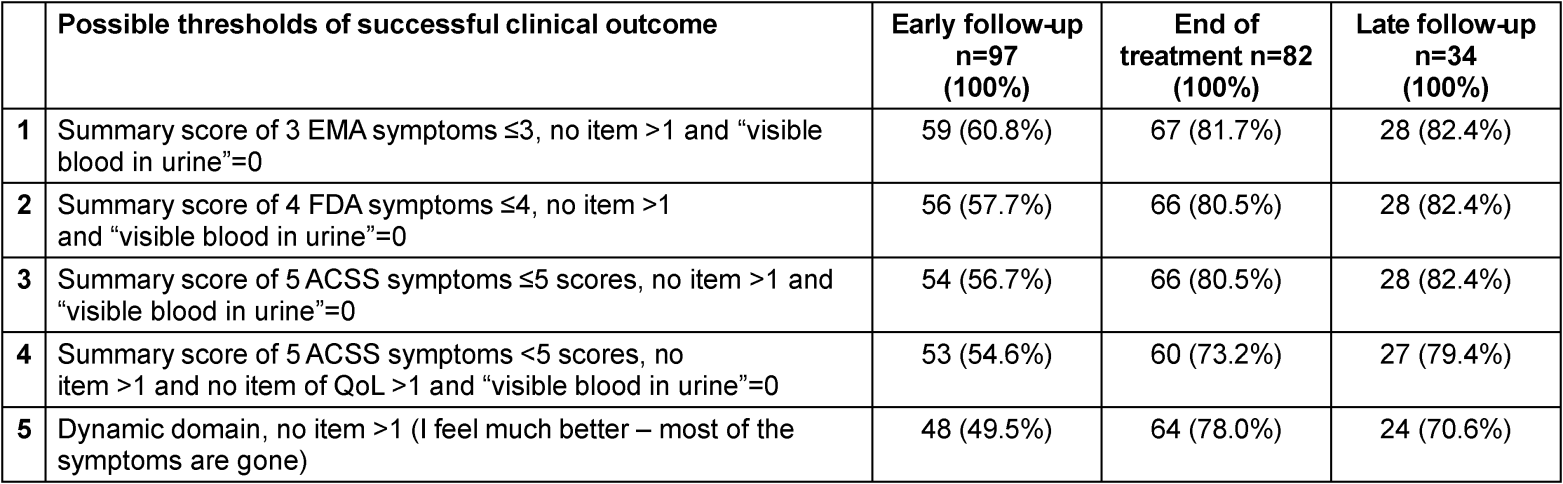
Successful clinical outcome according to different possible thresholds and at different follow-up [18]

**Table 3:**
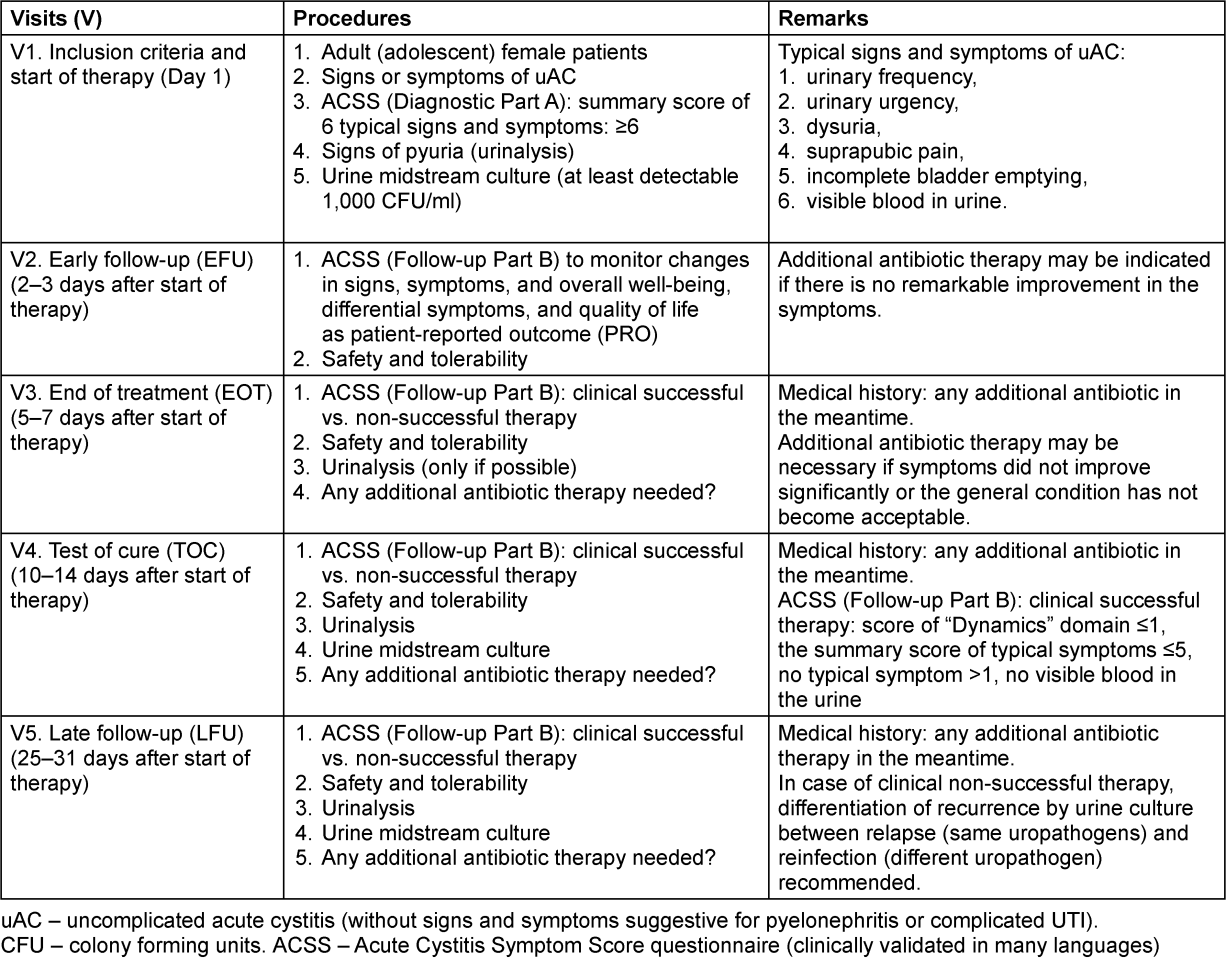
Proposed guidelines on the evaluation of non-antibiotic versus antibiotic agents indicated for the treatment of an acute episode of uncomplicated cystitis in adult (adolescent) female patients. Study design: prospective randomised, double-blind, placebo-controlled
