# Supplementary material for: Proposed guidelines on the evaluation of non-antibiotic versus antibiotic agents indicated for treatment of uncomplicated acute cystitis in adult female patients
Source: GMS Infect Dis. 2026 Jan 28;14:Doc04. doi: 10.3205/id000104 (PMC12914369; doi:10.3205/id000104)
Supplement: American English Acute Cystitis Symptom Score (ACSS) – Questionnaire [file ID-14-04-s-001.pdf]

Attachment 1: American English Acute Cystitis Symptom Score (ACSS) – Questionnaire

Adapted from: Alidjanov JF, Naber KG, Pilatz A, Wagenlehner FM. Validation of the American English Acute Cystitis Symptom Score. Antibiotics (Basel). 2020 Dec 19;9(12):929. DOI: 10.3390/antibiotics9120929
NB: Question 11 slightly modified

FIRST VISIT – Part A (diagnostic part) Time: : Date of evaluation: / / (mm/dd/yyyy)

Please indicate whether you have had the following symptoms during the past 24 hours, and how severe they were:

		(Please mark only one answer for each symptom)	0	1	2	3
Typical Symptoms	1	Frequent urination of small amounts of urine *(going to the toilet very often)*	☐ None *up to 4 times per day*	☐ Yes, mild *5–6 times/day*	☐ Yes, moderate *7–8 times/day*	☐ Yes, severe *9–10 or more times/day*
	2	Urgent urination *(a sudden and uncontrollable urge to urinate)*	☐ None	☐ Yes, mild	☐ Yes, moderate	☐ Yes, severe
	3	Feeling burning pain when urinating	☐ None	☐ Yes, mild	☐ Yes, moderate	☐ Yes, severe
	4	Feeling incomplete bladder emptying *(Still feel like you need to urinate after urination)*	☐ None	☐ Yes, mild	☐ Yes, moderate	☐ Yes, severe
	5	Feeling pain not associated with urination in the lower abdomen *(below the belly button)*	☐ None	☐ Yes, mild	☐ Yes, moderate	☐ Yes, severe
	6	Blood seen in urine *(without menses)*	☐ None	☐ Yes, mild	☐ Yes, moderate	☐ Yes, severe
				**Sum of "Typical" scores=**		**points**
Differential	7	Flank pain *(pain in one or both sides of the lower back)*	☐ None	☐ Yes, mild	☐ Yes, moderate	☐ Yes, severe
	8	Abnormal vaginal discharge *(abnormal amount, color and/or odor)*	☐ None	☐ Yes, mild	☐ Yes, moderate	☐ Yes, severe
	9	Discharge from the urethra *(urinary opening)* without urination	☐ None	☐ Yes, mild	☐ Yes, moderate	☐ Yes, severe
	10	a) Feeling high body temperature/fever<br>b) Temperature measured ☐ No ☐ Yes	☐ None (≤99.5˚F)	☐ Yes, mild (99.6˚F–100.2˚F)	☐ Yes, moderate (100.3°F–102.0°F)	☐ Yes, severe (≥102.1 ˚F)
				**Sum of "Differential" scores=**		**points**

Quality of Life

11 **Please indicate how these symptoms have interfered with your quality of life in the past 24 hours *(Please mark only one***

- ☐ 0 Did not interfere at all *(I feel as good as usual)*
- ☐ 1 Mildly interfered *(I feel a little worse than usual)*
- ☐ 2 Moderately interfered *(I feel much worse than usual)*
- ☐ 3 Severely interfered *(I feel terrible)*

12 **Please indicate how these symptoms have interfered with your everyday activities/work in the past 24 hours *(Please mark only one answer)*:**

- ☐ 0 Did not interfere at all *(Working as usual on a working day)*
- ☐ 1 Mildly interfered *(Due to the symptoms, I work slightly less)*
- ☐ 2 Moderately interfered *(Daily work requires effort)*
- ☐ 3 Severely interfered *(I almost cannot work)*

13 **Please indicate how these symptoms have interfered with your social activities *(visiting people, meeting with friends, etc.)* in the past 24 hours *(Please mark only one answer)*:**

- ☐ 0 Did not interfere at all *(My social activities did not change in any way, I live as usual)*
- ☐ 1 Mildly interfered (*Insignificant decrease in activities)*
- ☐ 2 Moderately interfered *(Significant decrease. I have to spend more time at home)*
- ☐ 3 Severely interfered *(It's terrible. I barely left the house)*

Sum of "QoL" scores= points

Additional

14 **Please indicate whether you have the following at the time of completion of this questionnaire:**

Menstruation (menses)?	☐ No	☐ Yes
Premenstrual syndrome *(PMS)*?	☐ No	☐ Yes
Signs of menopausal syndrome *(e.g. hot flashes)*?	☐ No	☐ Yes
Pregnancy?	☐ No	☐ Yes
Known (diagnosed) diabetes mellitus *(high sugar)*?	☐ No	☐ Yes

FOLLOW-UP Visit – Part B (patient-reported outcome) Time: : Date of evaluation: / / (mm/dd/yyyy)

Please indicate if you experienced any changes in your symptoms since the first time you completed this questionnaire

Dynamics

- ☐ 0 Yes, I feel back to normal (All symptoms are completely gone)
- ☐ 1 Yes, I feel much better (Most of the symptoms are gone)
- ☐ 2 Yes, I feel somewhat better (Only some symptoms are gone)
- ☐ 3 No, there are barely any changes (I still have about the same symptoms)
- ☐ 4 Yes, I feel worse (My condition is worse)

All questions of Part A, 1–14, follow here in Part B as well

Attachment 1 to: *Naber KG, Alidjanov JF, Pilatz A, Wagenlehner FM. Proposed guidelines on the evaluation of non-antibiotic versus antibiotic agents indicated for treatment of uncomplicated acute cystitis in adult female patients. GMS Infect Dis. 2026;14:Doc04.*
DOI: 10.3205/id000104, URN: urn:nbn:de:0183-id0001040
